# The Role of Intereukin-31 in Pathogenesis of Itch and Its Intensity in a Course of Bullous Pemphigoid and Dermatitis Herpetiformis

**DOI:** 10.1155/2017/5965492

**Published:** 2017-07-20

**Authors:** Lilianna Kulczycka-Siennicka, Anna Cynkier, Elżbieta Waszczykowska, Anna Woźniacka, Agnieszka Żebrowska

**Affiliations:** Department of Dermatology and Venereology, Medical University of Lodz, Poland Hallera Square No. 1, 90-647 Lodz, Poland

## Abstract

Itch which is one of the major, subjective symptoms in a course of bullous pemphigoid and dermatitis herpetiformis makes those two diseases totally different than other autoimmune blistering diseases. Its pathogenesis is still not fully known. The aim of this research was to assess the role of IL-31 in development of itch as well as to measure its intensity. Obtained results, as well as literature data, show that lower concentration of IL-31 in patients' serum may be correlated with its role in JAK/STAT signaling pathway which is involved in development of autoimmune blistering disease. Intensity of itch is surprisingly huge problem for the patients and the obtained results are comparable with results presented by atopic patients.

## 1. Introduction

Itch which occurs in a course of bullous pemphigoid (BP) and dermatitis herpetiformis (Duhring disease, DH) is a symptom which differentiates those two autoimmune blistering diseases among others. The reasons for which itch is present are still unknown.

Although the first definition of itch (unpleasant sensation leading to scratching) was made in 1660, its exact pathogenesis is still unknown. It is known that histamine, proteases, neuropeptides, acetylcholine, and bradykinin as well as receptors: opioid, cannabinoid, TRVP1 (transient receptor potential cation channel subfamily V member 1), and PAR2 (protease activated receptor 2) play an important role in the pathogenesis [1]. Moreover interleukins: IL-2, IL-8, and IL-31 are part of this response. In particular IL-31 is a subject of scientific interests in recent years. In most cases researches were made in patients with allergic diseases or atopy, particularly atopic dermatitis and prurigo [2–4]. Single reports present involvement of IL-31 in pathogenesis of itch in a course of other diseases [5–7]. So far there are no data connected with the role of IL-31 in development of itch in a course of autoimmune blistering diseases as well as itch intensity in a course of dermatitis herpetiformis. Moreover there are scarce data connected with itch intensity in a course of bullous pemphigoid [8, 9].

Interleukin-31 belongs to the family of IL-6 and is produced by Th2 lymphocytes. It works by activation of heterodimeric receptor which consists of two subunits: alpha (IL-31RA) and receptor for oncostatin M [2, 3]. Lymphocytes T (Th1, Th2, CD4+, and CD8+) as well as monocytes, macrophages, dendritic cells, mastocytes, eosinophils, and fibroblasts are the source of IL-31. In turn subunit A is localized on monocytes, eosinophils, dendritic cells, and keratinocytes.

Bullous pemphigoid is the most common autoimmune blistering disease which usually occurs among elderly patients [10, 11]. Pathogenesis of the disease is connected with presence of autoantibodies against antigens present in basement membrane: bullous pemphigoid antigen 2 (BPAG2, collagen XVII) whose molecular weight is 180 kD and bullous pemphigoid antigen 1 (BPAG1) with molecular weight 230 kD which are part of hemidesmosomes. It is also known that mediators excreted by mast cell play a role in development of the disease.

First symptoms of the disease may be unspecific. But itch is dominating symptom which is bothering patients. In a course of the disease many different clinical symptoms may be present: urticarial lesions, erythematous edema, papules, and eczematous lesions. Classic clinical picture of bullous pemphigoid is presence of tense blisters localized on normal-looking skin or on erythematous basis with coexistence of papules or erythema. Usually mucous membranes are not involved.

Dermatitis herpetiformis is more often present in young adult people but it is also the most common autoimmune blistering disease among children. Together with autoimmunological process against transglutaminases (TG) in the skin there is a silent or oligosymptomatic, gluten sensitive enteropathy. The reason for development of clinical symptoms in a course of the disease is still not fully understand process of formation of granular deposits of IgA in dermal papillae, as well as deposits of other immunoglobulins and complement components [12].

As in BP in a course of dermatitis herpetiformis polymorphic symptoms as papules, erythema, wheals and vesicles with herpetiform pattern, and rarely tense blisters are present. Moreover also secondary lesions which are result of scratching are possible. Typical localization on elbows, knees, buttocks, and scalp is characteristic. Skin symptoms are accompanied by very severe itch.

The aim of this paper was to evaluate role of IL-31 in development of itch in a course of bullous pemphigoid and dermatitis herpetiformis. Also intensity of itch was measured using proper questionnaires. Research was performed after acceptance of Bioethics Committee of Medical University of Lodz, Poland (RNN/145/09/KB-17.02.2009r).

## 2. Materials and Methods

### 2.1. Clinical Characteristic

The study was performed on 52 patients: 28 with bullous pemphigoid and 24 with dermatitis herpetiformis. Group of BP patients was composed of 21 women and 7 men, at the age of 49–91, mean 74.5. In turn group of patients with dermatitis herpetiformis was formed by 14 women and 10 men, at the age of 21–79, mean 41.9. The diagnosis of both diseases was made based on clinical pictures and positive results of direct immunofluorescence (DIF). Also indirect immunofluorescence (IIF) and skin biopsy were performed. In a group with pemphigoid additional test using salt split technique was made to exclude epidermolysis bullosa acquisita. At the time of examination all patients were in an active phase of the disease; survey questionnaires as well as blood samples were taken before treatment. Control group consisted of 13 healthy volunteers which are age and sex matched.

### 2.2. Measurement of IL-31 Concentration

To measure concentration of IL-31, 5 mL of blood was taken from both patients and control group. It was centrifuged for 10 minutes at 2500 rpm and frozen at −20°C. Concentration of IL-31 was measured in serum using ELISA and presented as mg/dL. Commercial BioLegend Kit was used. The assessment was made according to the producer's instruction. To get results calibration curves were established.

### 2.3. Measurement of Itch Intensity

Measurement of itch intensity was made in both groups: BP and DH. According to current rules two independent scales were used: four-item itch questionnaire and numeric rating scale (NRS).

The questionnaire prepared by Reich et al. [13] contains four questions which measure different aspects of itch: extent, intensity, frequency, and sleep disturbances. It is possible to get from 3 to 19 points where 3 means little intensity of itch and 19 means very severe intensity of itch. If there is no itch patient gets 0. Patients were asked to take into consideration only the latest 72 hours because the intensity of itch may be variable in a course of the disease.

The second tool is eleven-step rating scale. As an answer the patient gives the right number responding to the intensity of itch in a course of the disease. Zero means “no itch” and 10 means “the most intense itch ever.” According to the literature data proposed interpretation of obtained results by Polish population was made: 0: no itch, 1–3: mild itch, 3–7: moderate itch, 7–9: severe itch, and 9-10: very severe itch [14–16]. Because of ambiguous character of interpretation of extreme values we decided that patients who matched 3 were qualified as those with mild itch not moderate, those who matched 7 were qualified to be in the group “severe itch” not very severe, and those who matched 9 were qualified to be in the group “very severe itch.” Zero means no presence of itch. Similarly as in case of the questionnaire patients were asked to give answers taking into consideration only the latest 72 hours. Numeric rating scale is a variant of VAS (Visual Analogue Scale).

### 2.4. Statistical Methods

Results of IL-31 concentrations were analyzed taken into consideration differences among mean results obtained by BP, DH, and control group. Analysis of variance and NIR test as post hoc test were made. Differences at *p* < 0.05 were considered statistically significant.

Results obtained by using the questionnaire and NRS were analyzed using the nonparametric Mann–Whitney test and the *χ*^2^ test. Moreover to analyze differences between obtained results also the Pearson correlation (*r*) was used and its significance was checked by the Student *t*-test making for statistically important correlation linear regression equation.

All calculations were made using Statistica®10.

## 3. Results

### 3.1. IL-31 Concentration

**Figure 1:**
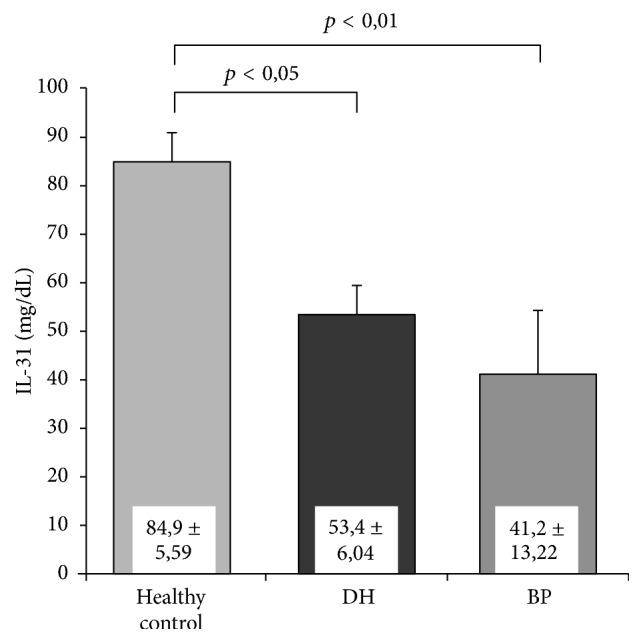
Mean IL-31 concentrations.

It was revealed that concentration of IL-31 was statistically significantly lower in both BP (41.2 ± 13.22; *p* < 0.01) and DH (53.4 ± 6.04; *p* < 0.05) patients in comparison with control group (84.9 ± 5.59) (Figure 1).

Nevertheless differences between patients' groups were statistically insignificant. Obtained results are shown in Table 1.

### 3.2. Itch Intensity

**Figure 2:**
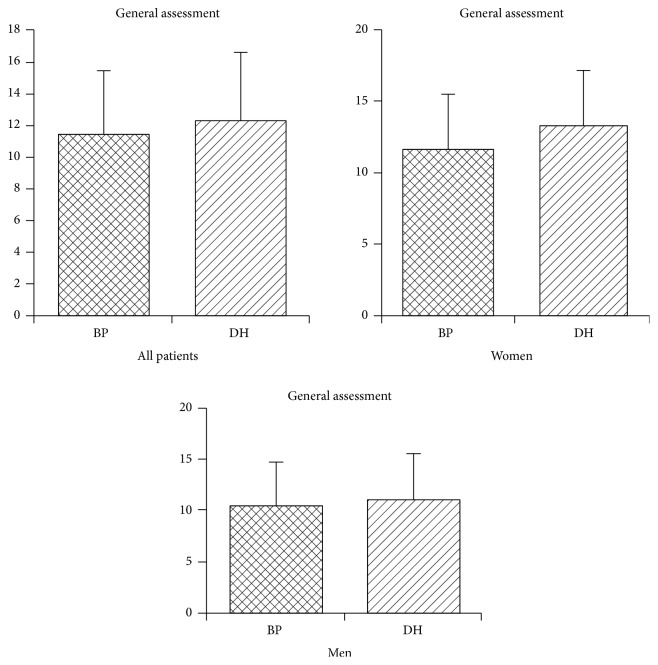
Itch intensity questionnaire, distribution of given answers.

Results achieved using itch intensity questionnaire showed that, for both groups of patients, with BP and DH, itch was similarly important problem. Patients with BP got from 5 to 19 points, mean 11.4. Women's results ranged from 5 to 19 points, mean 11.7. Men achieved from 6 to 18 points, mean 10.4. Presented differences were statistically insignificant (*p* > 0.05). Results obtained by DH patients were between 4 and 19 points, mean 12.4. Taking into consideration sex, women got from 5 to 19 points, mean 13.4, while men got from 4 to 19 points, mean 11.0. Presented differences were also statistically insignificant (*p* > 0.05).  Figure 2 shows distribution of answers given by both groups of patients.

In group with BP the most popular scores (for every score 4 out of 28 patients, which made 14.3%) were 7 and 16. In case of DH group most patients (5 out of 24, 20.8%) got 14 points. Answers given by patients with BP were more differentiated.

**Figure 3:**
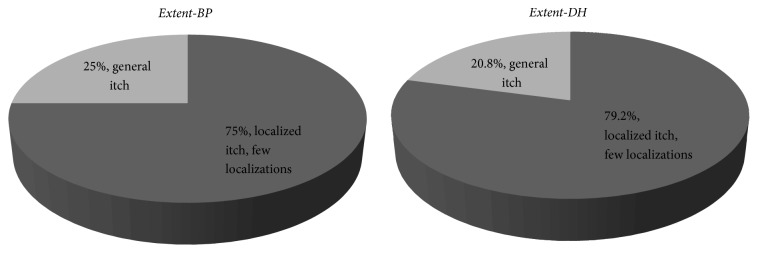
Itch extent, percentage of given answers.

Careful analysis of questions from itch intensity questionnaire showed that 75% of BP patients pointed that presence of itch is connected with a few localizations what means presence of skin lesions in particular area. Rest of the group pointed that their itch was generalized. Results obtained by DH patients were similar: 79.2% and 20.8%, respectively.  Figure 3 presents obtained results.

Taking into consideration intensity of itch 25% of DH patients showed general irritation because of that feeling. Similar percentage of patients revealed itch which provoked scratching with presence of excoriations as well as itch without relief after scratching, without presence of excoriations. Moreover, 12.5% of DH patients showed both: itch which needs scratching, without presence of excoriations, and presence of itch without need to scratch.

**Figure 4:**
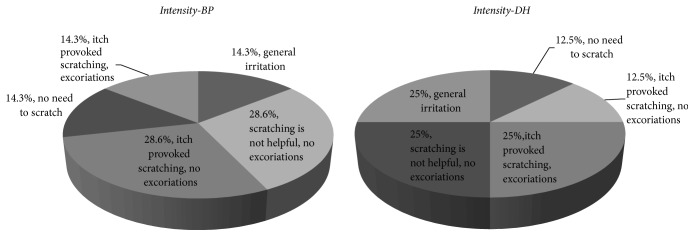
Itch intensity, percentage of given answers.

28.6% of patients with BP showed that their itch needed scratching without presence of excoriations. Next 28.6% of patients revealed that scratching is not helpful. In case of 14.3% of interviewees itch needed scratching with presence of excoriations and next 14.3% of patients showed that presence of itch is not connected with scratching.  Figure 4 presents the obtained results.

**Figure 5:**
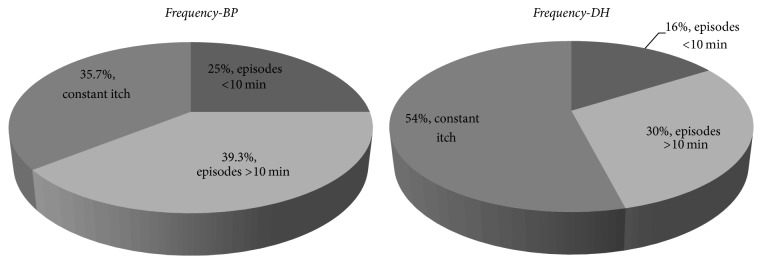
Itch frequency, percentage of given answers.

Most of the patients with DH (54%) reported itch as a constant feeling. 30% of patients with DH had episodes which last longer than 10 minutes and 16% shorter than 10 minutes. Only 35.7% of patients with BP reported itch as a constant problem, and not much more (39.3%) reported presence of episodes longer than 10 minutes. 25% of BP patients experienced itch which lasts no longer than 10 minutes. All results are presented on Figure 5.

**Figure 6:**
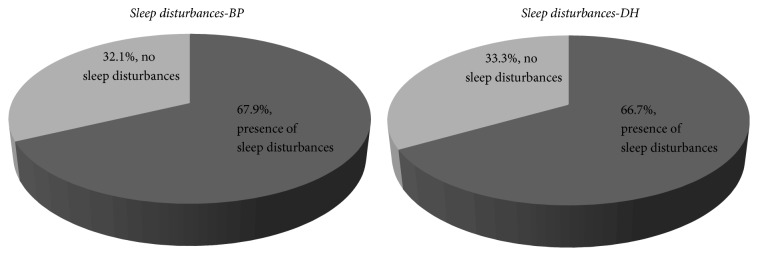
Sleep disturbances provoked by itch, percentage of answers.

Most of both DH patients (66.7%) and BP patients (67.9%) confirmed sleep disturbances provoked by presence of itch. In DH group 33.3% reported that they woke up many times during the night because of itching. Nevertheless the same amount of patients did not wake up at all. The rest of the interviewees woke up only once during night (12.5%) or twice (20.8%). By contrast patients with BP woke up twice during night (32.1%) or once (21.4%). Obtained results are presented on Figure 6.

Results obtained using four-item questionnaire are presented in Table 2.

### 3.3. Numeric Scale

It was shown that both groups of patients got similar results using numeric scale to assess itch intensity. The maximal number was 10 and it was pointed out by 25% of patients with BP and 20.83% of patients with DH. The minimal number was 4 and it was pointed out by 7.14% and 8.33%, respectively. Mean result was 7.7 in group with BP and 8.0 in group with DH.

Taking into consideration sex, women with BP marked from 4 to 10, mean 7.9, while men marked from 6 to 10, mean 7.3. In a group with DH obtained results according to sex were as follows: women from 7 to 10, mean 8.6, and men from 4 to 10, mean 7.3.

Also correlations between itch intensity questionnaire and results of NRS were assessed. In group of patients with BP statistically significant correlations (*p* < 0.0001) between results of NRS and general results of questionnaire (the whole results) and itch intensity and sleep disturbances were shown. In group of patients with DH statistically important correlations between NRS results and general intensity of itch (*p* < 0.001) and intensity of itch (one item from questionnaire) (*p* < 0.05) and sleep disturbances (*p* < 0.0001) were shown. In both investigated groups correlations between NRS results and itch extent as well as frequency were statistically insignificant (*p* > 0.05).

In group of patients with DH statistically significant correlations between general intensity of itch and particular items from questionnaire (*p* < 0.0001 for itch intensity and sleep disturbances, *p* < 0.001 for itch extent and frequency) were shown. In group of BP patients correlation between general itch intensity and itch extent was statistically insignificant (*p* > 0.05), while general itch intensity and other items from questionnaire revealed correlations were statistically significant (*p* < 0.0001). No statistically significant correlations between age of patients from both groups and general intensity of itch as well as particular items from the questionnaire and NRS results were shown. Also correlation between sex of patients from both groups and general intensity of itch as well as particular items of questionnaire and results of NRS were statistically insignificant (*p* > 0.05).

## 4. Discussion

Results of investigations conducted during past years, mainly among patients with atopic dermatitis, prurigo, chronic urticaria, and psoriasis, confirmed important role of IL-31 in pathogenesis of itch [2, 17]. No data are available about its role in autoimmune blistering diseases in course of which itch is also present as bullous pemphigoid and dermatitis herpetiformis.

We confirmed that concentration of IL-31 in serum from BP and DH patients is importantly lower than in a control group. This result is opposite to outcomes from research conducted in other diseases with presence of itch [2, 17]. Raap et al. showed higher concentration of IL-31 in serum from patients with atopic dermatitis [18] as well as spontaneous chronic urticaria [5]. In turn Narbutt et al. showed intensified expression of IL-31 in serum from patients with psoriasis [6]. After UVBNB exposure it becomes lower but did not achieve values as in a control group.

It is known that both bullous pemphigoid and dermatitis herpetiformis present in active phases Th2 cytokine profiles [19, 20]. Similar profile is in atopic dermatitis [21]. Our result suggests that IL-31 is a component of signal path responsible for itch development but dependsing on the disease paths may be different as well as the role of IL-31.

It is known that activated mastocytes contribute to higher expression of mRNA for IL-31 receptors [2, 3]. It is possible that hyperactivation of mastocytes which causes degranulation may be also responsible for higher expression of mRNA for IL-31 receptors, which may be the reason for low concentration of IL-31 in serum. Literature data show also that IL-31A receptors present on typical, human keratinocytes have different variants which depend on progress of cell division but also on impact of proinflammatory cytokines as INF*γ* [3]. Maybe in a course of bullous pemphigoid and dermatitis herpetiformis there is deposition of IL-31 in changed areas. The obtained result suggests the need to continue research especially taking into consideration assessment of IL-31 mRNA expression in skin biopsies from patients. That kind of research was conducted in a group of patients with atopic dermatitis, psoriasis, prurigo [22], and lichen planus [7]. Sonkoly et al. showed increased mRNA for IL-31 expression in more than half of the investigated patients with atopic dermatitis but not in case of psoriasis [22]. Similar observations were true for prurigo. Unfortunately they did not assess concentration of IL-31 in patients' serum. Also higher expression of IL-31 in skin biopsies from lichen planus patients was confirmed by Welz-Kubiak et al. without assessment of IL-31 concentration in patients' serum [7]. However intensity of itch was measured using two, independent scales—VAS and questionnaire containing twelve questions. It was shown that maximal intensity of itch was assessed by patients as medium (VAS max 6.5 ± 2.7) and at the time of assessment as mild (VAS 2.2 ± 1.8). Results obtained using questionnaire showed that patients got 6.9 ± 2.8 points. There was no correlation between IL-31 expression and itch intensity.

It is known that IL-31 acts also through JAK/STAT signaling pathway and activates JAK-1 and JAK-2 but also STAT-1, STAT-3, and STAT-5 [2]. Thus low concentration of IL-31 in patients' serum may be connected with involvement of IL-31 into the mentioned signaling pathway. Its involvement in pathogenesis of bullous pemphigoid and dermatitis herpetiformis needs future researches. Nevertheless literature data showed that IL-31 is rather responsible for itch induction compared to development of inflammatory skin lesions [17].

Assessment of itch intensity is difficult because of its subjective nature. There are many available methods but none is accepted as adequately objective and reliable. That is why two different, independent methods are accepted to assess intensity of itch.

We decided to use the questionnaire to assess intensity of itch because it was available and validated in Polish population and previously results were accessible [13]. On the other hand NRS, which is variant of VAS, is very easy to use and needs short time to fill. Literature data show that both scales may be used to assess itch intensity in clinical trials and the obtained results are comparable [23]. On this basis it is possible to accept equivalence of those two tools in assessment of pain intensity, which can be referring to evaluation of itch intensity. Nevertheless it is worth remembering that NRS is not free of defects and some of the patients have difficulties with understanding this type of tool. Moreover numeric scale does not give opportunity to statistical comparison with other, more descriptive tools. It may be due to different interpretation of particular terms and they may not be constant with those used by patients. Our results show that intensity of itch which is present in a course of BP and DH is from moderate to severe. Detection of positive correlation between results obtained using two different tools suggests their similar statistical value.

As it was mentioned before there are scarce data connected with itch intensity in a course of bullous pemphigoid [8, 9]. Bardazzi et al. showed that patients with BP suffered from moderate to severe pruritus [9]. To measure it they use 5-point Verbal Rating Scale (VRS), which is little bit different than scale used by us. That is why it is difficult to easily compare obtained results. Nevertheless in both cases itch is a serious problem for patients. Moreover Bardazzi et al. revealed a strong positive correlation between BP180 ELISA and VRS. Authors explained the result that BP180 ELISA is a monitoring instrument for BP, particularly in the assessment of itch. Also Kalinska-Bienias et al. showed that pruritus is an important problem for patients with BP [8]. Authors did not measure this symptom using separate tool but one of the questions from quality of life questionnaire takes into consideration this symptom.

Comparison of our results and literature data shows that itch in a course of BP and DH is only little bit weaker than in a course of atopic dermatitis. Chrostowska-Plak et al. showed that patients with atopic dermatitis got from 5 to 19 points, mean 14 [24]. In turn, intensity of itch assessed using VAS was 7.9 during last 2 weeks and 3.1 at the time of measurement. Mean value obtained by our patients with BP was 7.7 and with DH 8.0. Of course we are aware that our research has some limitations. First of all number of participating patients, especially with DH, is small. It is due to relatively rare occurrence of autoimmune blistering diseases. Moreover, we did not confirm relationship between patients' age and sex and obtained results. This is also consequence of small number of participating patients.

Mean results of NRS show that for our patients itch is an important problem. Comparison of the results with literature data demonstrates that more than one-third (37%) of atopic dermatitis patients also experience itch as a severe problem while only 8.1% as a very severe symptom [23]. It is quite surprising. It may be explained by subjectivity of the used method. Literature data confirm that age and sex as well as antihistamines (which are not effective in a course of autoimmune blistering diseases) have no influence on the obtained results.

## 5. Conclusion

To summarize, the role of IL-31 in pathogenesis of autoimmune blistering diseases is not fully known and that is why it needs future researches. Increasing knowledge in that field will be for sure helpful in development of new therapeutic methods, maybe less excessive than those available now. It is important especially for patients with bullous pemphigoid.

The consciousness that intensity of itch present in a course of both blistering diseases is comparable with symptoms reported by patients with atopic dermatitis, which is a model pruritic disease, gives us opportunity to develop right attitude to the patient. Furthermore it gives also opportunity to choose the right therapeutic strategy which takes into consideration not only skin improvement but also symptoms bothering patients. This is worth remembering as patient mental state stays in a strict relationship with compliance and effectiveness of treatment.

## Figures and Tables

**Table 1:** Results of variance analysis and NIR test as post hoc test for IL-31 in different groups.

	Control group	DH	BP
Control group		0.030925 (*p* < 0.05)	0.001662 (*p* < 0.01)
DH	0.030925 (*p* < 0.05)		0.358572 (*p* > 0.05)
BP	0.001662 (*p* < 0.01)	0.358572 (*p* > 0.05)	

**Table 2:** Results of itch intensity questionnaire.

	General assessment		Extent		Intensity		Frequency		Sleep disturbances	
	BP	DH	BP	DH	BP	DH	BP	DH	BP	DH
Mean	11.4	12.4	2.3	2.3	2.9	3.4	3.7	3.7	2.6	3.1
SD	4.1	4.3	0.4	0.4	1.2	1.3	1.4	1.7	2.1	2.5
SEM	0.77	0.88	0.08	0.08	0.23	0.27	0.26	0.35	0.40	0.51
MAX	19	19	3	3	5	5	5	5	6	6
Median	11	14	2	2	3	4	4	5	2	4
MIN	5	4	2	2	1	1	1	1	0	0
*n*	28	24	28	24	28	24	28	24	28	24

SD: standard deviation; SEM: standard error of the mean; *n*: number of patients.
